# Physiological responses and Ethylene-Response AP2/ERF Factor expression in Indica rice seedlings subjected to submergence and osmotic stress

**DOI:** 10.1186/s12870-023-04380-y

**Published:** 2023-07-27

**Authors:** Hsin-Yu Chi, Shang-Ling Ou, Mao-Chang Wang, Chin-Ying Yang

**Affiliations:** 1 International Master Program of Agriculture, National Chung Hsing University, Taichung, 402 Taiwan; 2 Department of Agronomy, National Chung Hsing University, Taichung, 402 Taiwan; 3 Department of Accounting, Chinese Culture University, Taipei, 111 Taiwan; 4 Smart Sustainable New Agriculture Research Center (SMARTer), National Chung Hsing University, Taichung, 402 Taiwan; 5 Innovation and Development Center of Sustainable Agriculture (IDCSA), National Chung Hsing University, Taichung, 402 Taiwan; 6 Advanced Plant and Food Biotechnology Center, National Chung Hsing University, Taichung, 402 Taiwan

**Keywords:** Ethylene, Antioxidant, Ethylene response factor, Alcohol dehydrogenase

## Abstract

**Background:**

The increased frequency of heavy rains in recent years has led to submergence stress in rice paddies, severely affecting rice production. Submergence causes not only hypoxic stress from excess water in the surrounding environment but also osmotic stress in plant cells. We assessed physiological responses and Ethylene-Response AP2/ERF Factor regulation under submergence conditions alone and with ionic or nonionic osmotic stress in submergence-sensitive IR64 and submergence-tolerant IR64-*Sub1* Indica rice cultivars.

**Results:**

Our results indicate that both IR64 and IR64-*Sub1* exhibited shorter plant heights and root lengths under submergence with nonionic osmotic stress than normal condition and submergence alone. IR64-*Sub1* seedlings exhibited a significantly lower plant height under submergence conditions alone and with ionic or nonionic osmotic stress than IR64 cultivars. IR64-*Sub1* seedlings also presented lower malondialdehyde (MDA) concentration and higher survival rates than did IR64 seedlings after submergence with ionic or nonionic osmotic stress treatment. Sub1A-1 affects reactive oxygen species (ROS) accumulation and antioxidant enzyme activity in rice. The results also show that hypoxia-inducible ethylene response factors (ERF)-VII group and *alcohol dehydrogenase 1* (*ADH1*) and *lactate dehydrogenase 1* (*LDH1*) genes exhibited different expression levels under nonionic or ionic osmotic stress during submergence on rice.

**Conclusions:**

Together, these results demonstrate that complex regulatory mechanisms are involved in responses to the aforementioned forms of stress and offer new insights into the effects of submergence and osmotic stress on rice.

**Supplementary Information:**

The online version contains supplementary material available at 10.1186/s12870-023-04380-y.

## Background

Rice (*Oryza sativa*) is the staple food of more than half the world’s population. In 2019, global rice production was 755.5 million tons; in the same year, rice production in Asia was 677.2 million tons, constituting 89.6% of global production and highlighting the importance of rice as a food crop in Asian countries [1]. However, climate change has led to an increased frequency of heavy rains, resulting in flooding that submerge rice paddies and severely affect production. Furthermore, almost all major rice-producing countries—including China, India, Thailand, and Indonesia—experience regular seasonal rainfall. Complete submergence of rice paddies during rainy seasons induces stress in rice crops, resulting in a drastic decline in rice production [2, 3].

Most dryland crops—such as barley, corn, sorghum, and wheat—can only withstand submergence stress for a short time [4]. By contrast, rice has well-developed gas spaces (i.e., aerenchyma) that enables it to survive for a long time under waterlogging conditions. Aerenchyma enables the continuous exchange of air between the roots and the aboveground parts of a plant, allowing oxygen to rapidly move from leaf tissue above water to root in hypoxic conditions. This mechanism increases the oxygen concentration in root cells, maintaining aerobic respiration [5, 6]. Sufficient oxygen supply to cells promotes the synthesis of NADPH and ATP followed by carbon dioxide fixation to form carbohydrates through dark reactions. Submergence not only lowers the exchange of oxygen and carbon dioxide but also hinders respiration and photosynthesis in plant cells, leading to the insufficient production of energy [7, 8]. Studies have demonstrated that in rice cells, stress caused by submergence induces the expression of sucrose synthetase in glycolysis pathways and the expression of pyruvic dehydrogenase and alcohol dehydrogenase in fermentation pathways [9]. Under hypoxic stress, lactic dehydrogenase activity increases in Arabidopsis species [10]. DNA microarrays were used to analyze gene expression in the M202 Sub1 and M202 rice cultivars submerged in water; different from M202 (the control group), M202 Sub1 exhibited the expression of sucrose synthetase, hexokinase, phosphofructokinase, aldolase, triose-phosphate isomerase, glyceraldehyde-3-phosphate dehydrogenase, and phosphoglycerate kinase in glycolysis pathways as well as pyruvate decarboxylase and acetaldehyde dehydrogenases in fermentation pathways [11].

When certain plants are completely submerged in water, excess water causes plant cell stomata to close, thereby reducing respiration and osmotic potential. Such submergence events result in hypoxic stress and osmotic stress [12, 13]. Osmotic stress can be further categorized as either nonionic or ionic. Nonionic stress (such as drought stress) strengthens a cellular osmotic potential, resulting in plant cell dehydration. Ionic stress causes toxic ion accumulation in cells. In plant cells, water is a medium for growth and metabolism, and the turgor pressure exerted by water supports cell walls. Water potential, either in the form of pressure (Ψp) or osmotic (Ψs) potential, alters the movement of water in cells, which affects the growth and metabolism pathways of such cells [14]. When crops encounter osmotic stress during their growth, such growth is affected and the crop yield is reduced [15]. Osmotic stress leads to plant dehydration, which results in the closing of the stomata, thereby restricting gas exchange. This chain of events inhibits metabolism and photosynthesis. Osmotic stress restricts gas exchange, resulting in hypoxic stress in plants and reduced respiration; under hypoxic stress, the energy metabolism pathways change, through the tricarboxylic acid (TCA) cycle, to an alcohol fermentation pathway as well as a lactic acid fermentation pathway [16–18].

Many studies have explored the biological responses to and molecular mechanisms underlying hypoxia caused by submergence stress [19–21]. However, few studies have examined the regulatory mechanisms of osmotic stress caused by submergence. Studies have indicated that submergence stress induces hypoxia and osmomotic stress [13]; the interaction between hypoxia and osmomotic stress are still poorly understood. therefore, to explain the biological characteristics and molecular regulation mechanisms of rice under submergence stress as well as ionic and nonionic osmotic stress, in this study, submergence-sensitive IR64 and submergence-tolerant IR64-*Sub1* rice cultivars were subjected to water submergence alone (Sub), submergence with mannitol (Sub + Man) or submergence with NaCl (Sub + NaCl). Our results presented that compared with IR64 seedlings, the IR64-*Sub1* seedlings exhibited a significantly lower plant height under Sub, Sub + Man, and Sub + NaCl treatments. IR64-*Sub1* seedlings had a lower malondialdehyde (MDA) concentration and higher survival rates than did IR64 seedlings after Sub + Man or Sub + NaCl treatment. Thus, Sub1A-1 affects reactive oxygen species (ROS) accumulation and antioxidant enzyme activity in rice. The results also show that hypoxia-inducible ethylene response factors (ERF)-VII group and *alcohol dehydrogenase 1* (*ADH1*) and *lactate dehydrogenase 1* (*LDH1*) genes exhibited complex regulation under nonionic or ionic osmotic stress during submergence.

## Results

### Growth differences in terms of plant height and root length in rice seedlings under submergence treatment alone and submergence treatment with nonionic or ionic osmotic stress

**Fig. 1:**
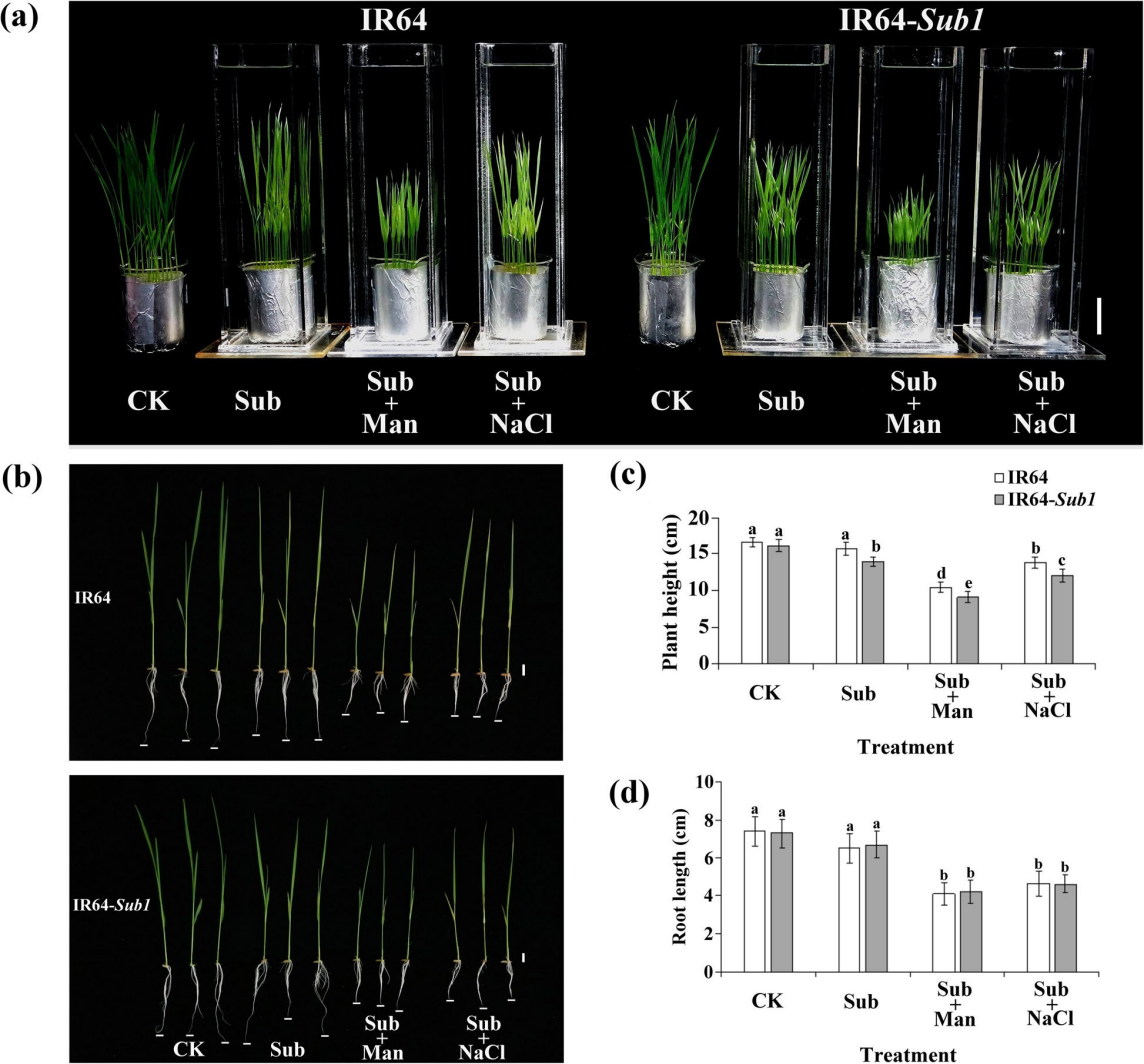
Physiological response of IR64 and IR64-*Sub1* seedlings subjected to submergence alone and submergence combined with nonionic or ionic stress. **a** The 8-day-old IR64 and IR64-*Sub1* seedlings were subjected to submergence (Sub), submergence with 300 mM mannitol (Sub + Man), submergence with 150 mM NaCl (Sub + NaCl) and control treatment (CK) for 8 days; scale bar = 5 cm. **b** Shoot and root phenotypes of IR64 and IR64-*Sub1* seedlings under various treatments for 8 days; scale bar = 1 cm. **c** The plant height and (**d**) root length were measured after Sub, Sub + Man, Sub + NaCl, or CK treatment. Average values ± SD (*n* = 30) are provided from data on three independent experiments. Different letters indicate significant differences between treatments and cultivars (*p* < 0.05 according to an LSD test using SAS)

**Fig. 2:**
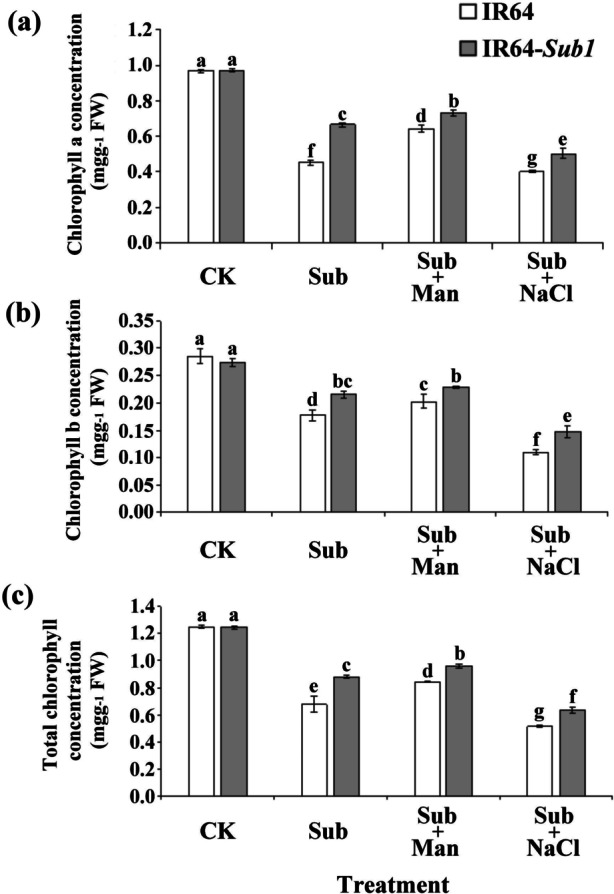
Chlorophyll concentration of IR64 and IR64-*Sub1* seedlings after different forms of osmotic stress under submergence treatment. The concentration of (**a**) chlorophyll a, (**b**) chlorophyll b and (**c**) total chlorophyll were measured after submergence (Sub), submergence with 300 mM mannitol (Sub + Man), submergence with 150 mM NaCl (Sub + NaCl) and control treatment (CK) for 8-day. The data represented average values ± SD from four biologically independent experiments. Different letters indicated significant differences between treatments and cultivars (*p* < 0.05 according to an LSD test using SAS)

To better understand the biological responses of rice seedlings to hypoxia combined with nonionic or ionic osmotic stress, we observed biological responses in IR64 and IR64-*Sub1* rice seedlings subjected to submergence (Sub), submergence with 300 mM mannitol (Sub + Man), submergence with 150 mM NaCl (Sub + NaCl), or control (CK) treatments. The results revealed that under a condition of submergence or submergence combined with osmotic stress, IR64 leaves exhibited a significantly paler yellow color than the IR64-*Sub1* leaves did, and after Sub + Man treatment, both IR64 and IR64-*Sub1* exhibited shorter plant heights and root lengths than did IR64 and IR64-*Sub1* subjected to Sub or Sub + NaCl treatment (Fig. 1a, b). The average plant heights of IR64 were 16.6, 15.7, 10.0 and 13.8 cm and those of the IR64-*Sub1* were 16.6, 13.9, 8.5 and 11.9 cm after CK, Sub, Sub + NaCl, Sub + Man treatment, respectively (Fig. 1c). No significant differences were observed in root length between the two cultivars under each treatment condition (Fig. 1d). An analysis of changes in the chlorophyll concentration of IR64 and IR64-*Sub1* rice under submergence stress and submergence with different forms of osmotic stress indicated that of the two cultivars, IR64 seedlings exhibited lower chlorophyll a, b, and total concentrations under submergence stress and submergence stress with different forms of osmotic stress. IR64 and IR64-*Sub1* displayed lower chlorophyll concentrations (a, b, and total) after Sub + NaCl treatment than after Sub or Sub + Man treatment (Fig. 2).

Further analyses of changes in the dry mass of rice seedlings under submergence stress indicated that the dry mass percentage of both IR64 and IR64-*Sub1* was significantly higher after Sub + Man treatment than that after submergence treatment alone (Table 1). This finding reveals that rice seedlings under submergence stress or submergence conditions with different forms of osmotic stress have different biological responses. Under a submergence condition with nonionic osmotic stress, although shorter plant heights and root lengths were observed, greater percentage of dry mass was noted.

**Table 1:** Fresh weight, dry weight and biomass percentage in 8-day old of IR64 and IR64-*Sub1* shoots under different osmotic stress during submergence

Treatment^*^	Fresh weight (mg)		Dry weight (mg)		Dry mass (%)	
	**IR64**	**IR64-*Sub1***	**IR64**	**IR64-*Sub1***	**IR64**	**IR64-*Sub1***
**CK**	69.8 ± 7.2^a**^	64.7 ± 4.2^ab^	10.0 ± 1.1^a^	9.1 ± 0.8^ab^	14.3 ± 0.5^bc^	14.1 ± 0.4^bc^
**Sub**	63.2 ± 2.2^b^	58.7 ± 3.2^bc^	..8.4 ± 0.3^bc^	8.5 ± 0.6^bc^	13.3 ± 0.1^c^	14.5 ± 0.8^b^
**Sub + Man**	41.8 ± 2.1^d^	40.5 ± 2.5^d^	..8.4 ± 0.5^bc^	8.3 ± 0.6^bc^	20.1 ± 0.3^a^	20.5 ± 0.2^a^
**Sub + NaCl**	56.5 ± 2.5^c^	55.1 ± 3.0^c^	..7.7 ± 0.7^bc^	8.1 ± 0.7^bc^	13.6 ± 1.4^bc^	14.7 ± 0.5^b^

^*^Treatments included submergence (Sub), submergence combined with 300 mM mannitol (Sub + Man), submergence combined with 150 mM NaCl (Sub + NaCl) and control check (CK)

^**^Different letters indicate significant differences between treatments (*p* < 0.05 depend on LSD test by Statistics Analysis System (SAS))

### Survival rates and redox equilibria of rice seedlings under submergence stress and submergence conditions with nonionic or ionic osmotic stress

**Fig. 3:**
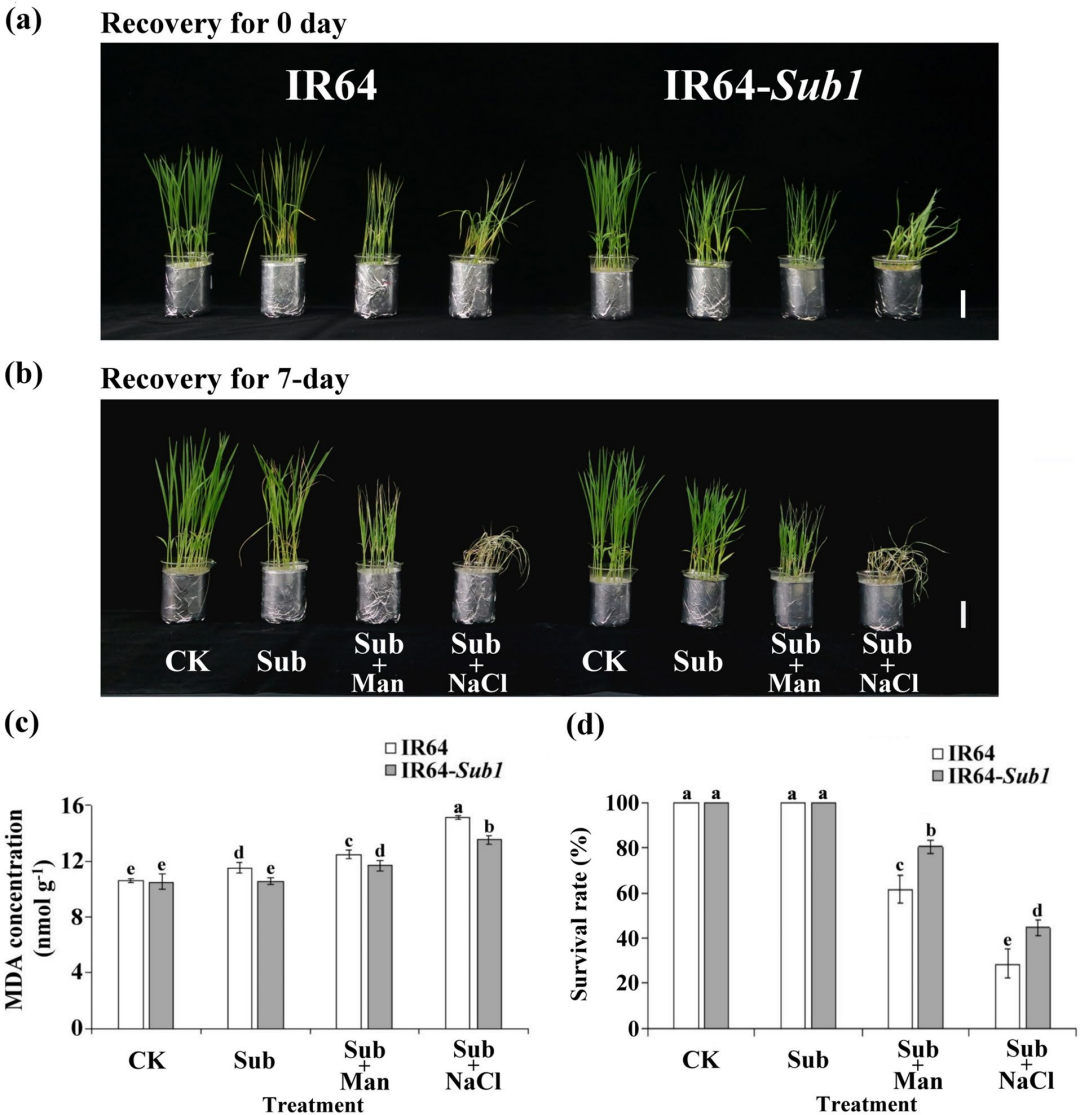
Concentration of malondialdehyde and its survival rate on IR64 and IR64-*Sub1* seedlings under submergence with and without nonionic or ionic stress. **a** Phenotypes of IR64 and IR64-*Sub1* seedlings after Sub, Sub + Man, Sub + NaCl or CK treatment for 8 days and (**b**) after subsequent recovery for 7 days. **c** Concentration of MDA after stress treatments. **d** Survival rates were evaluated after stress treatments. Average values ± SD are provided from data on three independent experiments. Different letters indicate significant differences between treatments and cultivars (*p* < 0.05 according to an LSD using SAS)

**Fig. 4:**
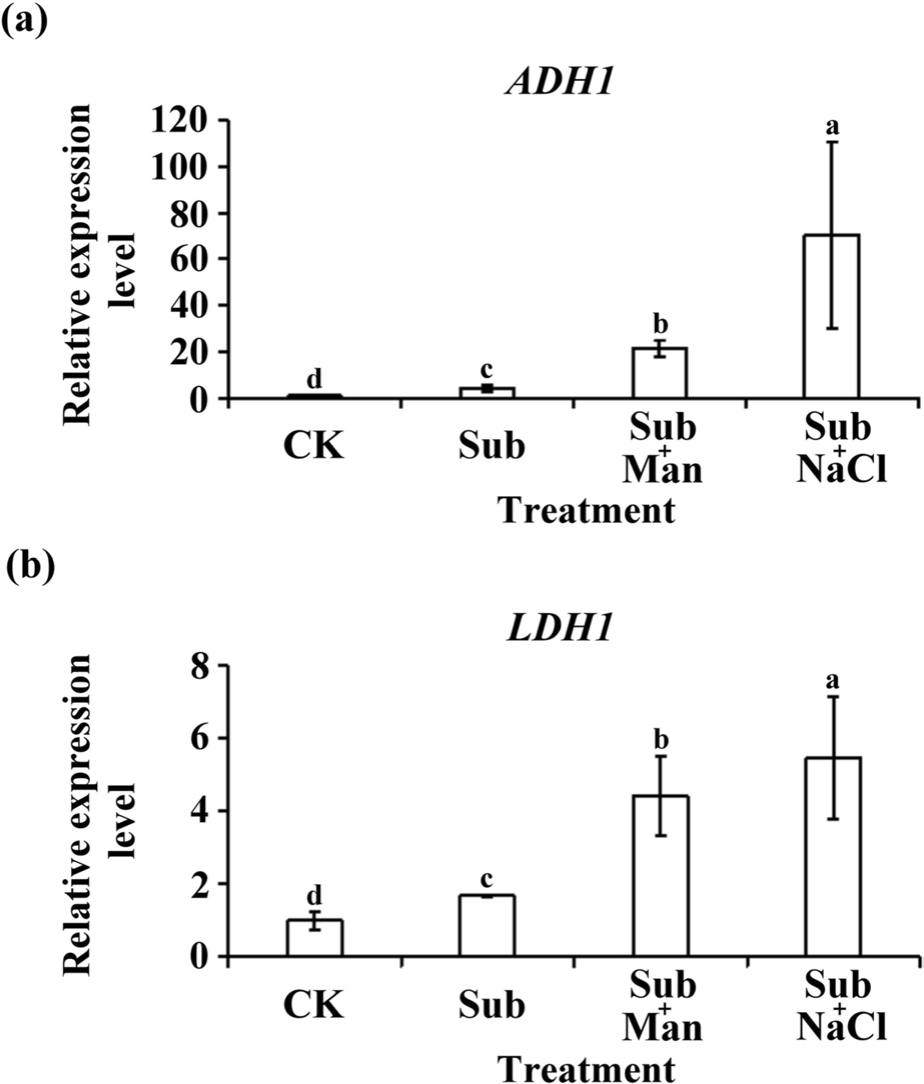
Transcript expression of hypoxia-inducible genes in IR64-*Sub1* seedlings after different treatments. Total RNA was isolated from shoots of 8-day old IR64-*Sub1* seedlings after submergence (Sub), submergence with 300 mM mannitol (Sub + Man), submergence with 150 mM NaCl (Sub + NaCl) and control treatment (CK) for 8-day, respectively. The expression levels of (**a**) *ADH1* and (**b**) *LDH1* mRNA were determined by qRT-PCR. The data represented average values ± SD from four biologically independent experiments. Different letters indicate significant differences between treatments and cultivars (*p* < 0.05 according to an LSD using SAS)

The IR64 and IR64-*Sub1* seedling survival rates were determined to further evaluate the effects of Sub, Sub + Man, Sub + NaCl, and CK treatments on rice seedlings. Both cultivars exhibited drooping after 8 days of Sub + NaCl treatment (Fig. 3a). After a 7-day recovery period, the survival rates of the seedlings were determined according to their ability to produce new leaves; the results indicated that after Sub + Man or Sub + NaCl treatment, the IR64-*Sub1* seedlings exhibited higher survival rates than the IR64 seedlings did (Fig. 3b, d), and the results of lipid peroxidation analyses indicated the IR64-*Sub1* seedlings had lower malondialdehyde (MDA) concentrations than the IR64 seedlings (Fig. 3c). The Sub, Sub + Man, and Sub + NaCl treatments induced the expression of hypoxia-inducible genes ADH1 and LDH1; the highest expression of such genes was observed after Sub + NaCl treatment (Fig. 4).

**Fig. 5:**
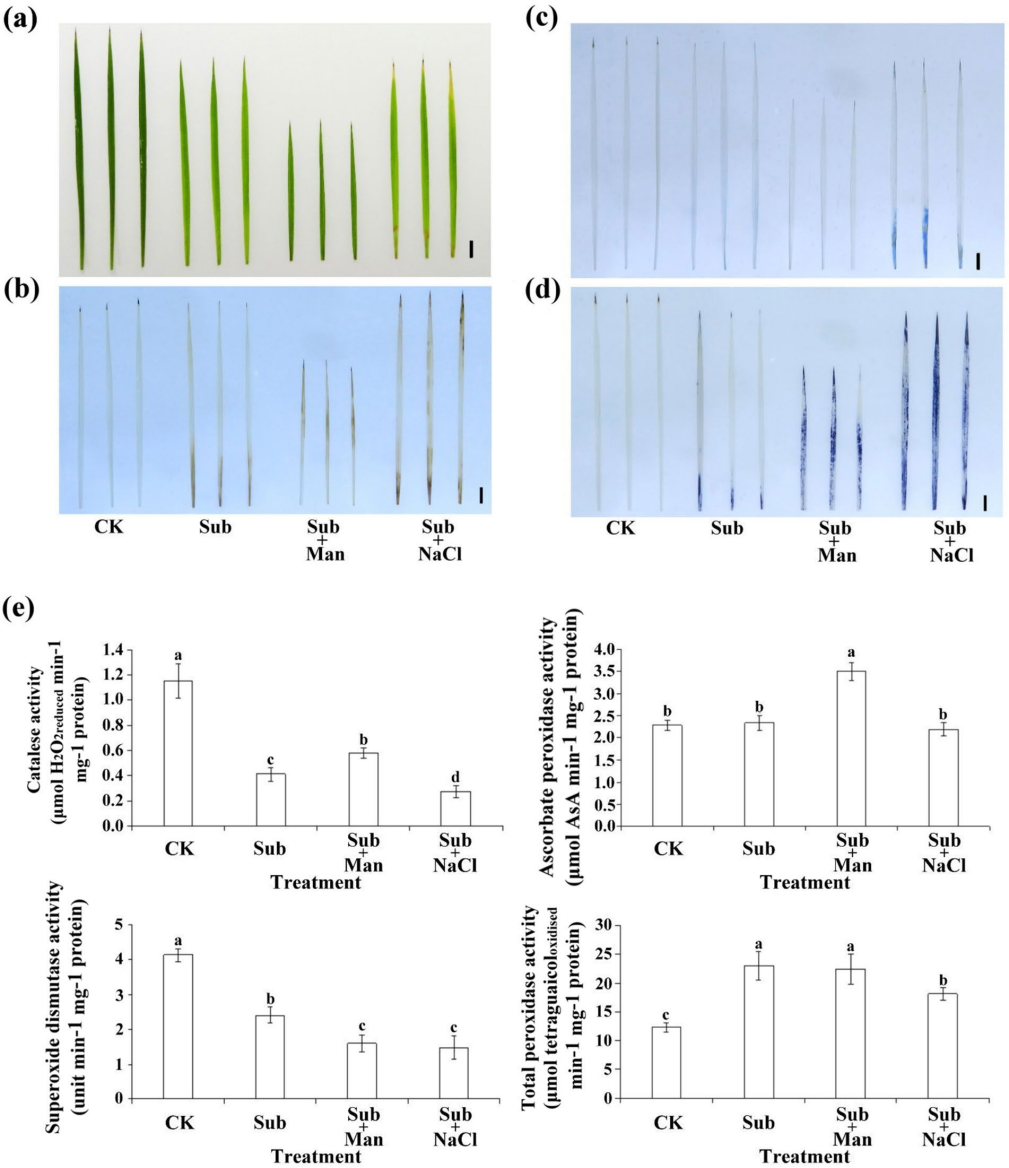
ROS accumulation, cell death, and antioxidant enzyme activity in the IR64-*Sub1* seedlings after stress treatments. **a** Phenotype of leaves in rice seedlings after different stress treatments. **b** DAB staining of hydrogen peroxide (H_2_O_2_). **c** NBT staining of superoxide (O_2_^−^). **d** Evans blue staining of dead cells. **e** Catalase (CAT), ascorbate peroxidase (APX), superoxidase dismutase (SOD), and total peroxidase (POD) activity assays. Scale bar = 1 cm. Three independent experiments were conducted

To further analyze whether Sub1A-1 affects ROS accumulation and antioxidant enzyme activity in rice, DAB and NBT tissue staining was used to detect H_2_O_2_ and O_2_^–^ in the rice leaves. The results revealed higher H_2_O_2_ accumulation in the tip and base of the leaves after Sub + NaCl treatment than after Sub or Sub + Man treatment (Fig. 5a–c). Furthermore, Evans blue staining revealed considerable leaf cell death after Sub + NaCl treatment (Fig. 5d). Antioxidant enzyme activity analyses demonstrated lower catalase (CAT) and superoxide dismutase (SOD) activity after Sub, Sub + Man, or Sub + NaCl treatment than after CK treatment; CAT activity was the lowest after Sub + NaCl treatment. High ascorbate peroxidase (APX) activity was only noted after Sub + Man treatment, and significantly increased total peroxidase (POD) enzyme activity was observed after treatment under each of the three stress conditions (Fig. 5e).

### Expression of ERF-VIIs in IR64-*Sub1* seedlings under submergence stress and submergence conditions with nonionic or ionic osmotic stress

**Fig. 6:**
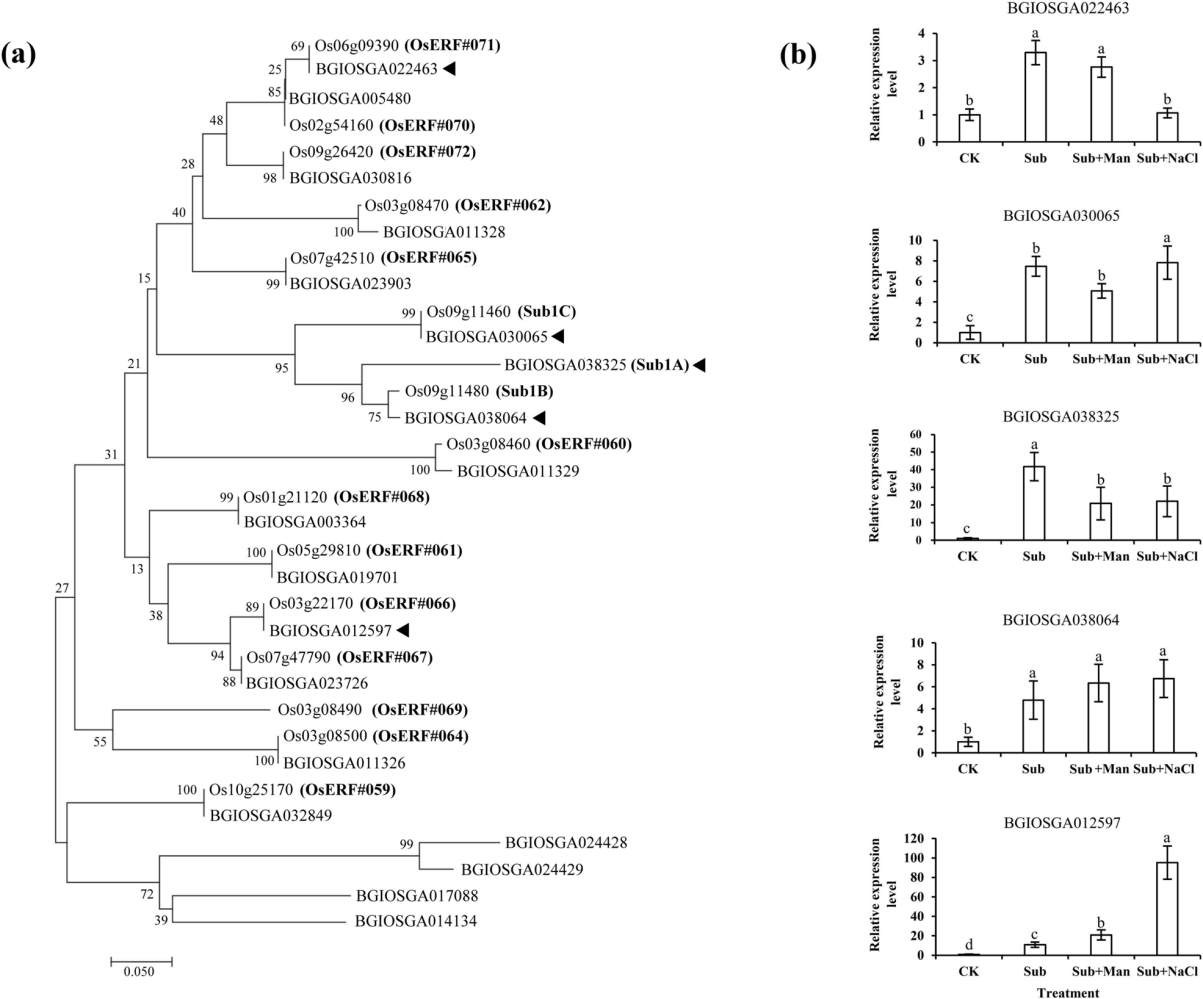
Phylogenetic tree and transcript expression of ERF-VII family genes in IR64-*Sub1* seedlings after various treatments. **a** Phylogenetic tree of japonica and indica rice ERF-VII proteins. **b** Expression levels of ERF-VII family gene mRNA were determined through qRT-PCR. The phylogenetic tree was constructed using the neighbor-joining method, and a summary of 1000 multiple bootstrapped replicates is shown on the nodes of the tree. The analysis was performed using MEGA 7 software. The sequence of *Oryza sativa* L. was obtained from the Plant Transcription Factor Database version 4.0. The qRT-PCR data represent average values ± SD from six independent experiments. Different letters indicate significant differences between treatments and cultivars (*p* < 0.05 according to an LSD test using SAS)

To further evaluate the classification of the ERF-VII family of *O*. *sativa japonica* and *O*. *sativa indica*, 15 *japonica* and 19 *indica* amino acid sequences from the Plant Transcription Factor Database version 4.0 (http://planttfdb.cbi.pku.edu.cn/index.php) were analyzed, and a phylogenetic tree was created (Fig. 6a). In *japonica* rice, five ERF-VII genes with water submergence–induced expression were selected from the phylogenetic tree (Fig. 6a). The homology genes in *indica* rice were BGIOSGA038325 (Sub1A), BGIOSGA038064 (Sub1B), BGIOSGA030065 (Sub1C), BGIOSGA022463, and BGIOSGA012597; the expression levels of such genes in IR64-*Sub1* seedlings after Sub, Sub + Man, Sub + NaCl, or CK treatment were evaluated using qRT-PCR. The results indicated that the expression of these five genes was also induced by submergence of the *indica* rice cultivar in water. Higher expression of BGIOSGA022463 was observed under submergence combine with nonionic stress than that under submergence combine with ionic stress, and the gene expression levels of BGIOSGA030065 and BGIOSGA012597 were significantly higher under submergence combine with ionic stress than under submergence combine with nonionic stress. No difference in the gene expression of BGIOSGA038325 or BGIOSGA038064 was observed under water submergence combine with ionic or nonionic stress (Fig. 6b).

## Discussion

Most related studies have focused on a single type of stress. However, in nature, the induction of only one form of stress is rare, and climate change has considerably increased the frequency of composite stress in plants. Numerous studies have demonstrated that during submergence, the diffusion of oxygen into plant cells is restricted, leading to hypoxic stress [22, 23]. Furthermore, rice seedlings with the Sub1A-1 gene have lower shoot elongation, which leads to higher biomass accumulation and resistance to submergence [24, 25]. However, submergence restricts plants’ use of oxygen. Furthermore, under submergence conditions, excess water in the surrounding environment may cause osmotic stress in plant cells, and the expression of Sub1A-1 genes may not be regulated by a single stress pathway [13]. As reported elsewhere, the shoot and root growth levels of Arabidopsis seedlings were restricted under osmotic stress, such as that induced by mannitol, sorbitol, or NaCl; under treatment with 300 mM mannitol, the aforementioned plants were observed to be tiny, dark green, and compact [26]. In this study, Indica rice cultivars IR64 and IR64-*Sub1* were subjected to Sub, Sub + Man, and Sub + NaCl treatments to compare the differences in physiological characteristics and molecular regulation as a result of different forms of osmotic stress under submergence conditions and to gain insight into mechanisms of osmotic regulation under such conditions. According to our observation, IR64-*Sub1* seedlings exhibited a significantly lower plant height under submergence conditions alone and with ionic or nonionic osmotic stress than IR64 cultivars. There could be various possible reasons and molecular mechanisms. One hypothesis is that the expression level of the Sub1 gene could be related to this phenomenon. The *Sub1* gene is a critical stress-responsive gene that exhibits significant differential expression under flooding and low oxygen stress conditions. This gene can increase the submergence tolerance of rice roots and reduce damage caused by root oxygen deficiency. Therefore, IR64-*Sub1* seedlings may exhibit lower plant height under submergence and osmotic stress conditions because they have a stronger response to these stressors. Additionally, other genes and pathways could also be involved in this phenomenon (Fig. 1). IR64-*Sub1* seedlings had a lower MDA concentration and higher survival rates than did IR64 seedlings after Sub + Man or Sub + NaCl treatment. Thus, Sub1A-1 affects ROS accumulation and antioxidant enzyme activity in rice (Figs. 3  and 5).

Flooding and submergence stress trigger changes in a plant’s genetic expression, which regulates a plant’s morphology and metabolism to adapt to stress [27]. Group VII ethylene response factors (ERF-VIIs) are a class of ERF transcription factors that are involved in adaptive responses to submergence and hypoxia through the regulation of the expression of multiple genes. In Arabidopsis, the five ERP-VII genes HRE1, HRE2, RAP2.2, RAP2.3, and RAP2.12 are key controlling factors of submergence or hypoxia tolerance; they strengthen plant tolerance to hypoxic stress through the regulation of hypoxia response genes. In rice, the ERF-VII transcription factor gene SUB1A has been recognized as a major controlling factor of submergence tolerance; it allows such plants to endure 14–16 days of complete submergence. ERF-VII factors SNORKEL1 and SNORKEL2 enable plants, especially deep-water crops, under submergence stress to escape their submerged environment through strengthened internode elongation. Studies have indicated the importance of ERF-VII in submergence responses and tolerance [28]. The results of the present study indicate that submersion induced the expression of ERF-VII genes BGIOSGA038325 (Sub1A), BGIOSGA038064 (Sub1B), BGIOSGA030065 (Sub1C), BGIOSGA022463, and BGIOSGA012597 in IR64-*Sub1* rice; furthermore, the genetic expression of BGIOSGA022463 was significantly higher after Sub + Man treatment than after Sub + NaCl treatment, whereas the genetic expression of BGIOSGA030065 and BGIOSGA012597 was significantly higher after Sub + NaCl treatment than after Sub + Man treatment (Fig. 6). ERF-VII genes may be involved in regulating complex signal transmission pathways while under submergence conditions with nonionic or ionic osmotic stress, and different regulatory mechanisms may occur under different forms of osmotic stress during submergence.

## Conclusions

Taken together, this study analyzed the physiological and molecular characteristics of IR64 and IR64-*Sub1* rice seedlings under submergence stress with and without nonionic or ionic osmotic stress. Because of the role of Sub1A-1, IR64-*Sub1* seedlings exhibited greater stress tolerance than did IR64 seedlings, allowing IR64-*Sub1* rice to adapt to different forms osmotic stress under submergence conditions. Furthermore, ERF-VIIs in rice had vital molecular regulatory mechanisms under different submergence and osmotic stress conditions.

## Materials and methods

### Plant materials, growth conditions and stress treatment

The rice cultivars used in this study were *indica* rice (*O*. *sativa*) IR64 and IR64-*Sub1*. The seeds were sterilized with 3% sodium hypochlorite for 30 min and then washed with sterile deionized water to remove sodium hypochlorite residue. The seeds were then placed in water at 37 °C in the dark to germinate for 1 day, after which the germinated seeds were placed on a Petri dish lined with damp filter paper to sprout for 1 day. Subsequently, the seeds were placed in a growth box at 28 °C with 16 h of illumination at a light intensity of 236 μmol m^−2^ s^−1^ and 8 h of darkness. After 2 days of culturing to germinate the seeds, the sprout seeds were moved onto a metal rack with beakers containing Kimura B solution [29]. Each beaker contained 25 seedlings, and the culture solution was replaced every 2 days until the seedlings were 8 days old, after which the seedlings were subjected to the experimental treatments (water submersion alone, Sub; submergence with mannitol; Sub + Man, and submergence with NaCl; Sub + NaCl). The seedlings in the control group (CK) were allowed to grow normally in an unsubmerged environment, and in the stress groups, the seedlings were placed in 7 cm × 7 cm × 30 cm water tanks, each simulating different types of osmotic stress. For the Sub group, the tank was filled with deionized water up to the 27-cm water level. For the Sub + Man group, the tank was filled with deionized water with 300 mM of mannitol up to the 27-cm water level. Finally, for the Sub + NaCl group, the tank was filled with deionized water with 150 mM sodium chloride up to the 27-cm water level. The seedlings and water tanks were placed in growth chamber for 8 days, and the aboveground parts of the plants were harvested as materials for subsequent analyses.

### Measurement of plant height, root length and chlorophyll content

IR64 and IR64-*Sub1* seedlings was treatments by Sub, Sub + Man, and Sub + NaCl and CK for 8-day, the shoot length and root length were measured for indicated times (at least 30 seedlings per treatment). The data was obtained from three biological repeats. For chlorophyll content assays as described in detail previously [30]. Chlorophyll a, b and total contents were extracted from 0.5 g of shoot tissue in 2 mL of sodium phosphate buffer (50 mM pH 6.8). The absorbance of the extraction was measured at A665 and A649 with a spectrophotometer (Metertec SP8001, Taiwan).

### Characterization and analysis of the survival rate and lipid peroxidation of the rice seedlings quantitative

The 8-day-old IR64 and IR64-*Sub1* seedlings were subjected to Sub, Sub + Man, or Sub + NaCl treatment for 8 days; subsequently, the plant height, root length, and the ratio of dry mass to fresh weight were measured. The height of each plant was measured from the base of the seedling to the tip of the leaves after the seedling leaves were straightened. The root length of a plant was measured by straightening the seedling roots and then measuring the length of the plant from the base to the tip of its roots. A plant’s fresh weight (FW) was measured by weighing the stress-treated seedlings, and a plant’s dry mass (DW) was determined by weighing it after it was dried at 60 °C for 48 h. At least three independent tests were performed, with 40 plants taken as samples in each independent test. Survival was determined by a plant’s ability to still grow new leaves after the stress treatment. The survival rates of IR64 and IR64-*Sub1* varieties were calculated by dividing the number of plants that were able to grow new leaves after 8 days of Sub, Sub + Man, or Sub + NaCl treatment and 7 days of recovery in a growth chamber by the total number of treated plants. The lipid peroxidation tests were conducted according to the approach of Hodges et al. [31], which involves measuring the malondialdehyde (MDA) concentration to analyze lipid peroxidation. The aboveground parts of the treated rice seedlings were harvested, ground into powder in the presence of liquid nitrogen, and mixed with a 5% (w/v) TCA buffer. The mixtures were centrifuged at 10,000 g and 20 °C, and the supernatant liquid was mixed with a TBA buffer [0.5 mL of 20% (w/v) trichloroacetic acid containing 0.65% (w/v) thiobarbituric acid]; after the solution was allowed to react at 95 °C for 60 min, it was immediately cooled in an ice bath. Finally, after centrifugation for 10 min at 3000 g, the A532 and A600 absorbance of the supernatant liquid was measured using a spectrophotometer (Metertech SP-8001, Taiwan). At least three independent tests were performed. The relevant formula is as follows:

$$\mathrm{MDA\;concentration }(\mathrm{nmol g}-1)\hspace{0.17em}=\hspace{0.17em}(\mathrm{A}532-\mathrm{A}600)/155\; (\mathrm{K},\mathrm{ mM}-1\mathrm{ cm}-1)\hspace{0.17em}\times \hspace{0.17em}5\;(\mathrm{reaction\;volume})\hspace{0.17em}\times \hspace{0.17em}4\;(\mathrm{dilution\;ratio})\hspace{0.17em}\times \hspace{0.17em}1000/\mathrm{FW }(\mathrm{g})$$

### Histochemical staining and antioxidative enzyme activity

The accumulation of H_2_O_2_ in cells was visualized using 3, 3′-diaminobenzidine (DAB) staining as previously described [32]. The NBT staining method detailed in Hückelhoven et al. [33] was adopted. First, NBT powder was dissolved in a solution of 10 mM tripotassium phosphate, and the solution was then distributed into centrifuge vials. After the 8-day-old rice seedlings were subjected to Sub, Sub + Man, or Sub + NaCl treatment for 8 days, their second leaves were harvested and soaked in NBT dye for 8 h in the dark. Thereafter, the dye was removed. The leaves were then placed in a boiling bath with 75% ethanol until the color of the leave was entirely faded. The Evans blue staining method of Tsai et al. [34] was adopted. After the 8-day-old seedlings underwent 8 days of Sub, Sub + Man, or Sub + NaCl treatment, the second leaves were harvested and soaked in a 0.1% Evans blue solution in the dark for 8 h. Independent tests were repeated at least three times.

To examine the antioxidant enzyme activity, the extracted samples first had to undergo the Bradford protein assay [35]. Catalase (CAT) activity was measured through grinding the aboveground part of the rice plant into a powder in the presence of liquid nitrogen in a prechilled mortar and then conducting analysis following the method of Kato and Shimizu [36]; 50 mM sodium phosphate buffer, which has a pH value of 6.8, was added to the powder in the prechilled mortar until the mixture was homogeneous. The mixture was centrifuged at 4 °C and 12,000 g, and the obtained supernatant liquid was the enzyme extract. After the Bradford assay was performed on the enzyme extract, 1 mg of protein was mixed evenly with 100 mM sodium phosphate buffer, which had a pH value of 7.0, and 1 M hydrogen peroxide. The changes in the absorbance of the supernatant were then monitored over 5 min using a spectrophotometer at a wavelength of 240 nm. The time used was 1 min. Each unit of enzyme activity represents the consumption of 1 μmol of hydrogen peroxide per minute. Total peroxidase (POD) activity was determined using the method of MacAdam et al. [37]; the aboveground part of the rice plant was ground into a powder in the presence of liquid nitrogen in a prechilled mortar. Lin and Kao [38] proposed the addition of potassium chloride (KCl) to a buffer solution to extract total peroxidase. In this experiment, a 50 mM potassium phosphate buffer, which had a pH value of 5.8 and contained 0.8 M KCl, was added to the prechilled mortar until the mixture was homogeneous; the mixture was then centrifuged at 4 °C and 12,000 g; the obtained supernatant liquid was the enzyme extract. The combined of 1 mg protein and 50 mM potassium phosphate buffer was mixed evenly with 21.6 mM guaiacol and 9 mM hydrogen peroxide, and changes in the absorbance of the solution was monitored over 5 min at a wavelength of 470 nm by using a spectrophotometer. The time used was 1 min. The control sample used comprised 99% alcohol. Each unit of enzyme activity represents 1 μmol tetraguaiacol produced per minute. One unit of superoxide dismutase (SOD) was defined as the amount of enzyme that inhibited by 50% the rate of β-NADH oxidation and absorbance was measured at A340 for 10 min with a spectrophotometer. For ascorbate peroxidase (APX) activity, the decrease in ascorbic acid (AsA) concentration was determined as the decline in absorbance at 290 nm and activity was calculated at 1 min by the extinction coefficient (2.8 mM^−1^ cm^−1^ at A290 nm) for AsA.

### Quantitative real-time PCR analysis

Samples were collected from seedlings and frozen until analysis. RNA was isolated from frozen tissues using TRIzol reagent (Invitrogen, Carlsbad, CA, USA). Total RNA samples were first treated with DNase I and then an MMLV First-Strand Synthesis Kit (Gene Direx, Grand Island, NY, USA) was used to synthesize the first strand of cDNA [reverse transcription (RT)]. Using membrane-anchored ubiquitin-fold (MUB) proteins as the internal control, this study performed quantitative real-time (qRT) PCR with specific primer pairs (Supplementary Table 1). Reactions were conducted using a Bio-Rad real-time PCR detection system (CFX ConnectTM, Bio-Rad, USA), and relative expression was analyzed using Bio-Rad CFX Manager 3.1 software (CFX ConnectTM, Bio-Rad). The ubiquitin gene was used as an internal control to normalize the cDNA levels. The reaction conditions were 94 °C for 5 min and 45 cycles at 94 °C for 30 s, 60 °C for 30 s, and 72 °C for 30 s. Independent tests were performed at least three times, and the results were averaged. The sequences of primers used for qRT-PCR are presented in the supplementary information (Additional file 1).

## Supplementary Information

**Additional file 1.** Primers used for quantitative RT-PCR experiments.

## Data Availability

Data sharing not applicable to this article as no datasets were generated or analysed during the current study.

## Abbreviations

SUB: Submergence
ROS: Reactive oxygen species
H_2_O_2_: Hydrogen peroxide
MDA: Malondialdehyde
ADH1: Alcohol dehydrogenase 1
LDH1: Lactate dehydrogenase 1
CAT: Catalase
SOD: Superoxide dismutase
APX: Ascorbate peroxidase
POD: Total peroxidase
ERF: Ethylene response factors
