# Supplementary material for: Physiological responses and Ethylene-Response AP2/ERF Factor expression in Indica rice seedlings subjected to submergence and osmotic stress
Source: BMC Plant Biol. 2023 Jul 27;23:372. doi: 10.1186/s12870-023-04380-y (PMC10373351; doi:10.1186/s12870-023-04380-y)
Supplement: Supplementary file 1 — Additional file 1. Primers used for quantitative RT-PCR experiments. [file 12870_2023_4380_MOESM1_ESM.docx]

Supplement table 1 The primer sequence of real-time PCR used in this study.

| *Indica* rice ID | *Japonica* rice ID | Gene name | Forward sequence (5’→3’) | Reverse sequence (5’→3’) |
| --- | --- | --- | --- | --- |
| BGIOSGA011163 | LOC_Os03g13170 | Ubiquitin | 5’-aaccagctgaggcccaaga-3’ | 5’-acgattgatttaaccagtccatga-3’ |
| BGIOSGA034312 | LOC_Os11g10480 | ADH1 | 5’-gcaaatttctggctttgtcaatcagta-3’ | 5’-cgccaaaagatcactgattcttaacaa-3’ |
| BGIOSGA007342 | LOC_Os02g01510 | LDH1 | 5’-agaaggcttcgtctctgtcg-3’ | 5’-ccgatcaccgatatcttcgt-3’ |
| BGIOSGA012597 | LOC_Os03g22170 | ERF66 | 5’-gtacggacagagccaagagg-3’ | 5’-catcgaaattccacatgagc-3’ |
| BGIOSGA022463 | LOC_Os06g09390 | ERF71 | 5’-tcagagatcctcgcaaaggt-3’ | 5’-taagctctggcagcttcctc-3’ |
| BGIOSGA038325 |  | Sub1A | 5’-cttcttgctcaacgacaacg-3’ | 5’-aggctccagatgtccatgtc-3’ |
| BGIOSGA038064 | LOC_Os09g11480 | Sub1B | 5’-gtttccatgttcccttctgg-3’ | 5’-acctctgtcgtcgtctccat-3’ |
| BGIOSGA030065 | LOC_Os09g11460 | Sub1C | 5’-tactcatcgagtgctgctcc-3’ | 5’-tagctccagaagcgcatgtc-3’ |
